# Prior Low- or High-Intensity Exercise Alters Pacing Strategy, Energy System Contribution and Performance during a 4-km Cycling Time Trial

**DOI:** 10.1371/journal.pone.0110320

**Published:** 2014-10-20

**Authors:** Carlos Rafaell Correia-Oliveira, Ralmony Alcantara Santos, Marcos David Silva-Cavalcante, Romulo Bertuzzi, Maria Augusta Peduti Dal’Molin Kiss, David John Bishop, Adriano Eduardo Lima-Silva

**Affiliations:** 1 Sports Science Research Group, Department of Physical Education and Sports Science, CAV, Federal University of Pernambuco, Vitória de Santo Antão, Pernambuco, Brazil; 2 Endurance Performance Research Group, School of Physical Education and Sport, University of São Paulo, São Paulo, São Paulo, Brazil; 3 Nephrology Division, Department of Medicine, Federal University of São Paulo, São Paulo, São Paulo, Brazil; 4 Institute of Sport, Exercise and Active Living, College of Sport and Exercise Science, Victoria University, Melbourne, Victoria, Australia; University of Rome Foro Italico, Italy

## Abstract

We analyzed the influence of prior exercise designed to reduce predominantly muscle glycogen in either type I or II fibers on pacing and performance during a 4-km cycling time trial (TT). After preliminary and familiarization trials, in a randomized, repeated-measures crossover design, ten amateur cyclists performed: 1) an exercise designed to reduce glycogen of type I muscle fibers, followed by a 4-km TT (EX-FIB I); 2) an exercise designed to reduce glycogen of type II muscle fibers, followed by a 4-km TT (EX-FIB II) and; 3) a 4-km TT, without the prior exercise (CONT). The muscle-glycogen-reducing exercise in both EX-FIB I and EX-FIB II was performed in the evening, ∼12 h before the 4-km TT. Performance time was increased and power output (PO) was reduced in EX-FIB I (432.8±8.3 s and 204.9±10.9 W) and EX-FIB II (428.7±6.7 s and 207.5±9.1 W) compared to CONT (420.8±6.4 s and 218.4±9.3 W; P<0.01), without a difference between EX-FIB I and EX-FIB II (P>0.05). The PO was lower in EX-FIB I than in CONT at the beginning and middle of the trial (P<0.05). The mean aerobic contribution during EX-FIB I was also significantly lower than in CONT (P<0.05), but there was no difference between CONT and EX-FIB II or between EX-FIB I and EX-FIB II (P>0.05). The integrated electromyography was unchanged between conditions (P>0.05). Performance may have been impaired in EX-FIB I due a more conservative pacing at the beginning and middle, which was associated with a reduced aerobic contribution. In turn, the PO profile adopted in EX-FIB II was also reduced throughout the trial, but the impairment in performance may be attributed to a reduced glycolytic contribution (i.e. reduced lactate accumulation).

## Introduction

To date, the effects of reduced endogenous carbohydrate (CHO) stores on performance during a time trial (TT) has received limited attention [1]–[4]. A study by Rauch et al. [1] reported that three days on a high-CHO diet resulted in an increase in the mean power output (PO) during a 60-min cycling TT compared to a control-CHO diet. This increase in PO occurred as early as the first minute of exercise. Despite significantly different starting muscle glycogen values and TT performances, it is interesting to note that the CHO-loaded condition enabled the participants to finish the trial with the same critical low muscle glycogen level as the control-CHO condition [1]. Recently, a reduction in overall performance (∼11%) was observed during a middle-distance cycling TT (200 kJ, ∼12 min) carried out after a validated protocol designed to lower muscle glycogen content, when compared to a control condition [3]. This reduction in performance was accompanied by a shorter fast-start, followed by a less pronounced increase in PO during the last part of the trial. The authors attributed the impaired PO at the end to a reduced glycogenolytic activity. Indeed, the final sprint seems to occur as a result of the spared muscle glycogen and anaerobic capacity in the earlier stages of a TT, accompanied by a consequent increase in the anaerobic energy expenditure at the end of the trial [3], [5], [6]. Together, these results suggest that pacing during a cycling TT is affected by pre-exercise muscle glycogen availability.

While it seems clear that pre-exercise muscle glycogen availability affects the pacing strategy, it is not known if fiber-selective glycogen depletion will have different effects on pacing strategy. Due to the high-intensity at the beginning of a middle-distance cycling TT [7], type II fibers may have a greater contribution initially [8]–[10]. However, since type II fibers are quickly fatigable, a decline in PO after a fast start may be associated with a reduced contribution from type II fibers [11], preserving the limited anaerobic energy reserve of type II fibers to support the increase in PO in the last part of the trial [5]. These specific changes in muscle fiber recruitment pattern during a typical middle-distance TT have not been investigated, probably due to limitations of the gold-standard muscle biopsy technique. Since this technique can increase variability and interfere directly with athletes’ pacing strategy and performance, the utilization of more practical and applied protocols has been recommended [12].

An interesting and practical manner to test the effects of fiber-selective glycogen depletion on pacing strategy would be to use validated protocols to reduce selectively the muscle glycogen content of type I or II fibers [8]–[10], and to verify if this affects pacing strategy and performance during a cycling TT. It has been shown that prolonged exercise lasting ∼3 h predominantly reduces the muscle glycogen stores from type I fibers [8], [10], while high-intensity, intermittent exercise reduces the muscle glycogen from type II fibers [9], [10]. Furthermore, the results would have important practical implications since many athletes participate in multi-stage races in which they perform a middle-distance TT a few hours after either prolonged or short cycling races.

Therefore, the aim of this study was to analyze the influence of prior selective muscle glycogen-lowering exercise, designed to predominantly reduce the muscle glycogen content of either type I or II fibers, on pacing strategy and performance during a 4-km cycling TT. We hypothesized that the exercise protocol designed to predominantly lower the muscle glycogen content of type I fibers would impair PO during the midpoint of the TT, while the exercise protocol designed to predominantly lower the muscle glycogen content of type II fibers would impair PO at the beginning and during the last part of the TT. In order to provide some insights about muscle recruitment [13], we also measured the muscle EMG signal throughout the TT.

## Methods

### Ethics Statement

This study was approved by the Ethics Committee of the Federal University of Alagoas.

### Participants

Ten amateur, competitive cyclists [age 33±2 years, mass 74.4±3.3 kg, height 173.7±2.4 m, body fat 12.3±1.6%, peak oxygen uptake (VO_2peak_) 4.1±0.1 L·min^−1^ (55.3±2.6 mL·kg^−1^·min^−1^), peak PO (PPO) 253.5±8.6 W] took part in this study. The athletes trained regularly (4–5 days per week) for more than 60 minutes per day and cycled more than 200 km per week. The sample size required was estimated, using mean power output (i.e. performance) as the main outcome, from the equation n = 8e^2^/d^2^, as suggested by Hopkins [14], where *n*, *e*, and *d* denote predicted sample size, coefficient of variation, and the magnitude of the treatment effect, respectively. Coefficient of variation was assumed to be 1.9% [15] and a detection of a very conservative 2% difference as statistically significant would require at least 7 participants. All study procedures, as well as protocol, benefits, and risks were explained before the commencement of the trials, and participants gave their written informed consent.

### Experimental Design

Participants reported to the laboratory on seven different occasions. During the first visit, participants answered a questionnaire on readiness for physical activity (PAR-Q), and underwent anthropometric measures [16] and an incremental test to establish their VO_2peak_ and PPO [17]. At the second visit, participants performed a 4-km cycling familiarization trial. The subsequent visits (visits 3 to 7) were performed in a randomized and repeated-measures crossover design. In the evening before the main trial (∼7PM), participants performed either an exercise protocol designed to predominantly reduce muscle glycogen content in type I fibers (EX-FIB I) [10], or type II fibers (EX-FIB II) [10], or no exercise (CONT). Participants then followed a standardized diet and performed in the next morning (∼7 AM) a 4-km TT. A 1-week interval was applied between the experimental sessions to wash out any residual effect of fatigue. The tests were performed at the same time of the morning to minimize circadian variation [18]. Participants performed all tests (incremental and TTs) on a provided racing bike attached to a stationary cyclosimulator (Trainer Flow Ergo T1680, Tacx, Wassenaar, Netherlands). Before each trial, the cyclosimulator was calibrated in accordance with the manufacturer’s recommendations. To date, the reliability and accuracy of the specific cyclosimulator model used in the present study has not been reported, although this has previously been provided in a similar cyclosimulator from the same manufacturer [19]. Participants were asked to refrain from vigorous physical activity, alcohol, tobacco, and caffeine for 24 hours before each experimental session. Subjects were blinded to the objectives of this study until all tests had been performed.

### Incremental test

The seat was adjusted vertically and horizontally for each participant and cycling shoes were used to secure the feet to the pedals. The seat position was recorded and replicated during all subsequent experimental trials. Participants performed an incremental test starting with a 3-min warm-up at 100 W. After the warm up, 30 W increments were applied every minute until the participant could no longer maintain the required pedal frequency between 80–90 rev·min^−1^. Measures of VO_2_, carbon dioxide production, and ventilation were continuously sampled throughout the test and averaged over 30-s intervals using an on-line breath-by-breath gas analyzer (Quark CPET, Cosmed, Rome, Italy). VO_2peak_ was defined as the average VO_2_ during the last 30 s of the test. PPO was determined as the highest PO achieved during the last completed 60-s stage. When participants were not able to complete the last stage, the PPO was calculated from the following equation:
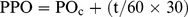
(1)where PO_c_ is the PO in watts of the last completed stage performed by the participant, t is the time (in seconds) sustained during the last incomplete stage, and 30 corresponds to the increments in PO in watts at each stage.

### Familiarization trial

The gear ratio was standardized at the beginning of the TT (i.e., 53×16), but participants were free to adjust the gear and pedal frequency once the TT had started. The participants were instructed to perform the TT in the shortest possible time and to remain seated throughout test. During the 48 h before the trial, the athletes were instructed to record all foods consumed (type, amount and time). These registers were later used to standardize the diet before the experimental trials. The validity of the diet register has been described in previous studies [20].

### Exercise protocols to reduce muscle glycogen content

For the three conditions, participants were asked to replicate exactly the diets recorded before the familiarization session during the morning and afternoon of the muscle-glycogen-reducing exercise day (total energy intake ∼2400 kcal, 60% CHO, 20% protein, and 20% fat). Participants arrived at the laboratory in the evening (between 6∶00 and 8∶00 PM), at least two hours after their last meal. Then, they either cycled at an intensity corresponding to 30% of their PPO (75.9±2.6 W) for 3 h or performed 10 sets of 1 min at a PO corresponding to 120% of their PPO (303.8±10.3 W), interspersed with 5-min rest periods. These protocols were designed to reduce predominantly the muscle glycogen stores from either type I fibers (evening before EX-FIB I trial) [8], [10] or type II fibers (evening before EX-FIB II trial) [9], [10], respectively. These protocols were validated by Carter et al. [10] using biopsies of the vastus lateralis muscle. They showed that after an exercise at 30% PPO for 3 h, approximately 94% of type I fibers had their muscle glycogen depleted, while just 30 and 4% of the type IIa and IIx fibers, respectively, were depleted. On the other hand, after an exercise at 120% PPO, approximately 99% of type II fibers (type IIa and IIx) were depleted, while only 15% of the type I fibers had their muscle glycogen reduced. These results have also been corroborated by others [8], [9].

Following the muscle-glycogen-reducing protocols (∼10∶00 PM), the participants were instructed to consume a low-CHO dinner (total energy ∼480 kcal, 13% CHO, 26% protein, and 61% fat) provided by the investigators and instructed to not consume any other additional food. For the control trial, participants were asked to consume a normal-CHO dinner (total energy intake ∼480 kcal, 67% CHO, 13% protein, and 20% fat), but no exercise was carried out in the prior evening.

### Experimental trials

Experimental tests were performed the next morning (between 8∶00 and 10∶00 AM) in a stable laboratory environment (22.9±1.1 °C and 44.9±2.9% relative humidity). Two hours before the trials, participants consumed a standardized breakfast, either with ∼67% CHO, 13% protein, and 20% fat (∼680 kcal) for the CONT condition (equal to amount usually consumed as registered in the recall), or containing a low CHO content (∼680 kcal, 13% CHO, 26% protein, and 61% fat) for both EX-FIB I and EX-FIB II conditions. A reduced CHO breakfast was administered during EX-FIB I and EX-FIB II conditions to guarantee that muscle glycogen would not have been replenished [21]. Maximal voluntary contractions were carried out immediately before each TT. Thereafter, the participants performed a 5-min warm-up at 100 W followed by a 5-min recovery. Then, a 4-km cycling TT was completed as fast as possible. Feedback about elapsed distance was given every 200 m, but participants were not informed about PO, velocity or cadence.

### Measurements

VO_2_, carbon dioxide production, ventilation, and respiratory exchange ratio (RER) were measured breath-by-breath during all experimental trials. Participants used a mask with ventilation and gas exchange responses measured via a computerized system (Quark CPET, Cosmed, Rome, Italy). The gas analyzer was calibrated according to the manufacturer’s specifications before each test (Quark CPET instruction manual) using room air and a standard gas mixture (16% oxygen and 5% carbon dioxide). The ventilometer was calibrated with a 3-L syringe. The PO was measured every 1 second (Tacx Trainer software 3.0, Wassenaar, Netherlands).

25 µL of blood were drawn from the ear lobe at rest, immediately before (Pre-TT), and 1 min after the TT (Post-TT). The blood samples were transferred into micro-tubes containing sodium fluoride (1%) and then centrifuged at 3000 rpm at 5°C for 10 minutes. Plasma lactate concentrations [La] were measured through enzymatic colorimetric reactions in a spectrophotometer (546 nm wavelength, model Q798U2V5, Quimis, São Paulo, Brazil).

To obtain the EMG signal, the skin of the vastus lateralis muscle of the right leg was shaved, abraded, and cleansed with isopropyl alcohol. Then, bipolar surface silver-silver chloride electrodes (Hal, São Paulo, Brazil), at a 20-mm interelectrode distance, were placed over the muscle belly and aligned to the muscle fiber direction. Before the TT, four 5-s submaximal isometric contractions of the knee (50%, 60%, 70% and 80% of the participant's subjective maximum) were performed as a warm-up [22]. Thereafter, the maximal voluntary contraction and corresponding EMG signal were obtained. Participants carried out three 5-s isometric maximal voluntary contractions of the knee extensors of both legs at an angle of 60° (full knee extension being the 0°), with a 1-min rest between them [23]. Participants were verbally encouraged during each maximal voluntary contraction. The peak force during the three contractions was measured by a load cell (EMG System Brazil, São Paulo, Brazil). The EMG signal of vastus lateralis muscle during each maximal voluntary contraction, as well as during each TT (every 200 m), was captured during 5-s intervals with a sampling frequency of 2000 Hz (Four-channel electromyograph, model 410C, EMG System Brazil, São Paulo, Brazil). Raw EMG signals were full-wave rectified and, to remove external interference noise and movement artifacts, filtered with a second-order, Butterworth, band-pass filter with cut-off frequencies set at 10 and 400 Hz. The integrated EMG (iEMG) was normalized by dividing the value obtained during the TT (at each 200 m) by the iEMG obtained during the highest maximal voluntary contraction [22], [23]. The procedures were performed using MATLAB 7.5 software (Mathworks Inc., Natick, US).

### Calculations

The PO corresponding to aerobic and anaerobic contribution (P_aer_ and P_an_, respectively) were estimated from calculations using the metabolic power and gross mechanical efficiency, in accordance with Hettinga et al. [24]. First, the P_met_ was calculated from the following equation [25]:
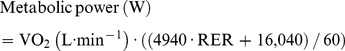
(2)where VO_2_ is the oxygen uptake in L·min^−1^ and RER is the respiratory exchange ratio. During the warm-up for each experimental trial, the gross mechanical efficiency was determined using the following equation:
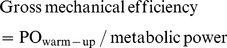
(3)where PO_warm-up_ is the PO in the warm-up (i.e., 100 W). As gross mechanical efficiency was not different across the conditions, the values were averaged for subsequent analyses. The P_aer_ during the TT was calculated by the equation:
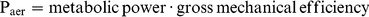
(4)where P_aer_ is the aerobic mechanical power output. An RER equal to 1.00 was assumed during the TT to calculate P_met_ [24]. The P_an_ was calculated from the following equation:
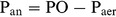
(5)where P_an_ is the anaerobic mechanical power output, PO is the power output and P_aer_ is the aerobic power output.

### Statistical analysis

Data distribution was verified by Shapiro-Wilk’s test. Means of the performance time, PO, P_aer_, P_an_, iEMG, and maximal voluntary contraction were compared between experimental conditions using repeated-measures ANOVA. When a significant effect was found, the main effect was analyzed using the least significant difference test for pairwise comparisons. A linear mixed model with Bonferroni correction was used to verify the effects of condition and distance on dependent variables (PO, P_aer_, P_an_, iEMG and [La]). To allow magnitude-based inferences about practical effects of prior low or high-intensity exercises on 4-km cycling time-trial performance, the uncertainty in the effects were expressed as likelihoods that the true value of the effects represent substantial changes (harmful, trivial or beneficial) [26]. The smallest standardized change was assumed to be 0.20. Data are expressed as means and standard error of the mean (mean ± SEM). All analyses were carried out in SPSS (13.0) software and the statistical significance was accepted at p<0.05.

## Results

### Maximal voluntary contraction

The maximal voluntary contraction carried out immediately before each TT was not significantly different between the three experimental conditions (CONT: 1332.5±80.1, EX-FIB I: 1265.5±74.2 and EX-FIB II: 1305.1±59.1 N; P>0.05), indicating that there was no influence (residual effect of muscle fatigue) from the exercise protocol performed in the evening before the TT.

### Time trial performance, and mean PO, P_aer,_ P_an_ and iEMG

The means for performance time, PO, P_aer_, P_an_, and iEMG are shown in Table 1. The time to complete the TT was significantly slower in EX-FIB I (P = 0.001) and EX-FIB II (P = 0.004) than in CONT, but there was no difference between EX-FIB I and EX-FIB II (P = 0.344). Using magnitude-based inferences, the prior low- and high-intensity exercise revealed a 98.5 and 92.6% chance that the true value of the effect statistic is ‘very likely’ and ‘likely’ harmful to performance, respectively. Similarly, mean PO was lower in EX-FIB I (P = 0.001) and EX-FIB II (P = 0.025) than in CONT, but there was no difference between EX-FIB I and EX-FIB II (P = 0.705). Likewise, the corresponding qualitative inferences were 98.3 and 86.2% ‘very likely’ and ‘likely’ harmful to performance, respectively. The mean P_aer_ in the EX-FIB I was significantly lower than in the CONT (P<0.05), but there was no difference between CONT and EX-FIB II or between EX-FIB I and EX-FIB II (P>0.05). Mean P_an_ and iEMG were similar for all conditions (P>0.05).

**Table 1:** Mean performance time, power output, aerobic and anaerobic power output, and integrated electromyographic activity during the 4-km cycling time trial in the control and reduced muscle glycogen conditions.

	CONT	EX-FIB I	EX-FIB II
Time (s)	420.8±6.4	432.8±8.3*	428.7±6.7*
PO (W)	218.4±9.3	204.9±10.9*	207.5±9.1*
P_aer_ (W)	163.8±6.0	155.1±7.0*	157.5±5.9
P_an_ (W)	54.6±6.6	49.8±6.5	50.0±5.8
iEMG (%)	50.5±2.1	53.3±1.7	45.8±1.3

Values are means ± SEM. PO: power output. P_aer_: aerobic power output. P_an_: anaerobic power output. iEMG: integrated electromyographic activity expressed as a percentage of their EMG value obtained during the maximal voluntary contraction. CONT: control condition. EX-FIB I and EX-FIB II: time trial performed after a carbohydrate-manipulation protocol designed to predominantly reduce muscle glycogen content in type I or II fibers, respectively. * Significantly different from CONT (P<0.05).

### Pacing strategy

**Figure 1:**
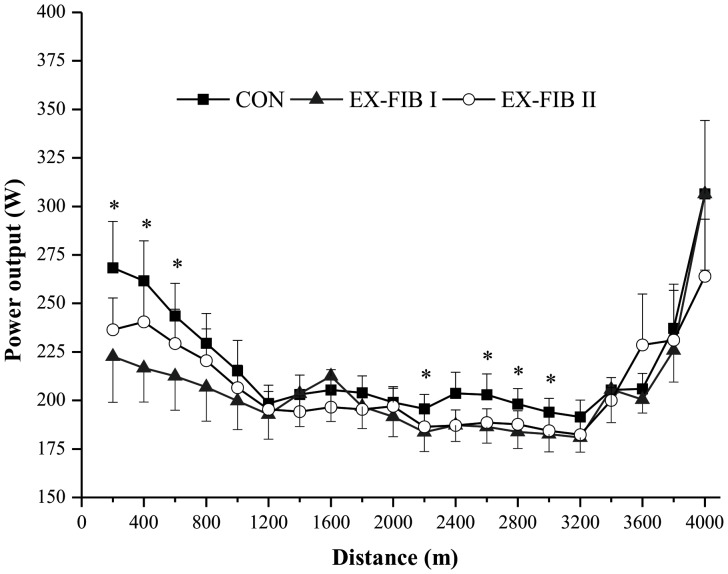
Mean and SEM for power output during the 4-km cycling time trial in the control (CONT) and reduced muscle glycogen conditions. EX-FIB I and EX-FIB II: time trial performed after a type I or type II muscle fibers glycogen-lowering exercise protocol, respectively. * CONT significantly higher than EX-FIB I for the same interval (P<0.05).

There were significant main effects for condition (P<0.05), distance (P<0.05), and interaction effects (P<0.05) for PO (figure 1). In the EX-FIB I condition, participants adopted a more conservative pacing strategy at the beginning and middle of the trial, when compared with CONT (P<0.05). The PO during the first 400 m and last 200 m was significantly higher than several points in the middle of the TT for CONT, but remained relatively stable up to 3.8 km in both EX-FIB I and EX-FIB II (P>0.05), when there was a significant increase in the last 200 m (figure 1).

**Figure 2:**
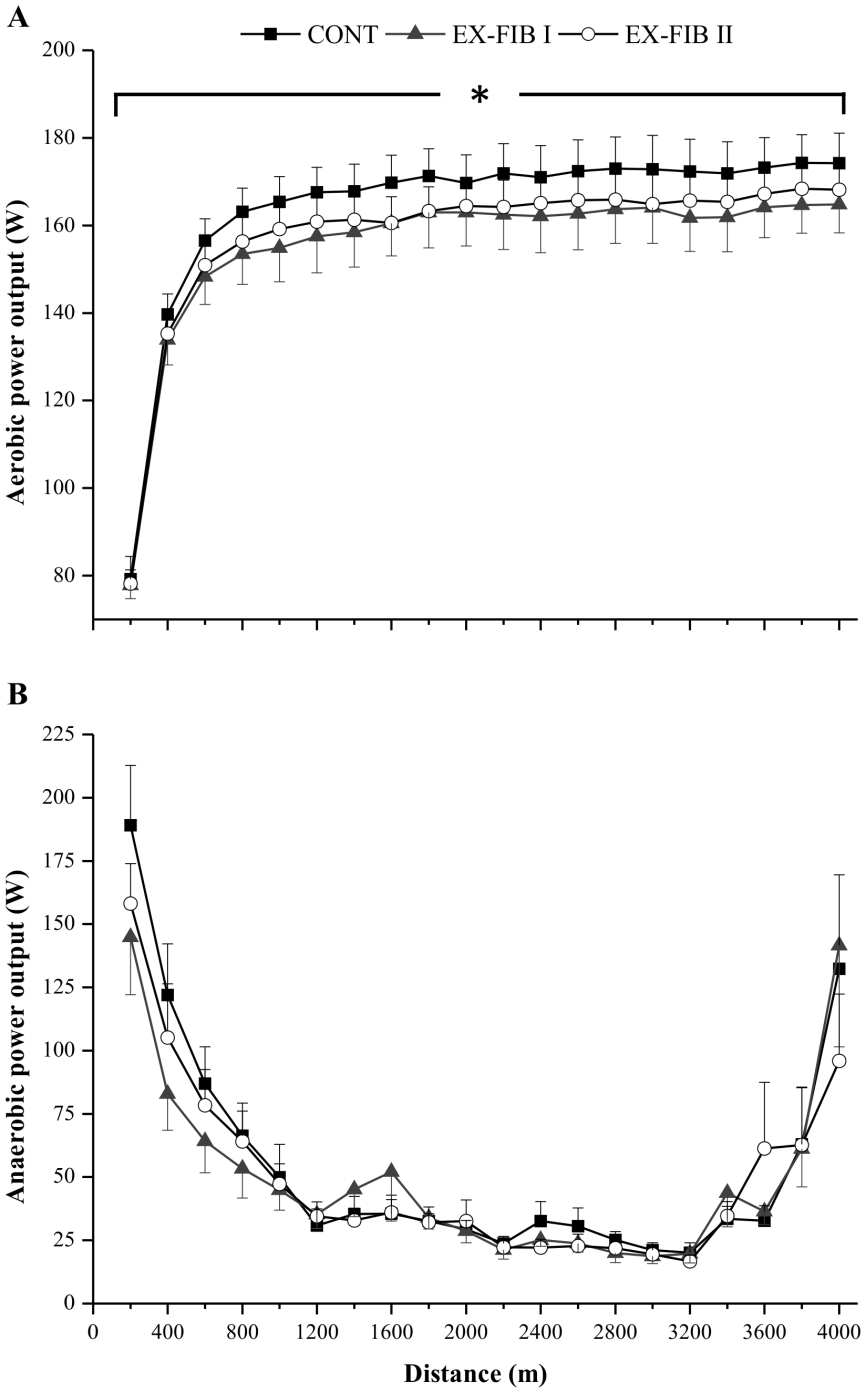
Mean and SEM for aerobic (A) and anaerobic (B) contribution during the 4-km cycling time trial in control (CONT) and reduced muscle glycogen conditions. EX-FIB I and EX-FIB II: time trial performed after a type I or type II muscle fibers glycogen-lowering exercise protocol, respectively. (A) * Significantly main effect of condition (P<0.05).

The results concerning the P_aer_ and P_an_ every 200 m are shown in Figures 2A and 2B, respectively. Main effects for condition and distance were found for P_aer_ (P<0.05), but there was no significant interaction (P>0.05). In all conditions, the P_aer_ increased exponentially and stabilized after the first 800 m (P<0.05), but was systematically lower during EX-FIB I than in the control (P<0.05).

There was also a main effect for P_an_ only for distance (P<0.05), but no condition (P>0.05) or interaction effect (P>0.05). In general, the P_an_ in the first 600 m and last 200 m was higher than the other intervals for all conditions (P<0.05; figure 2B).

### iEMG and Plasma lactate

**Figure 3:**
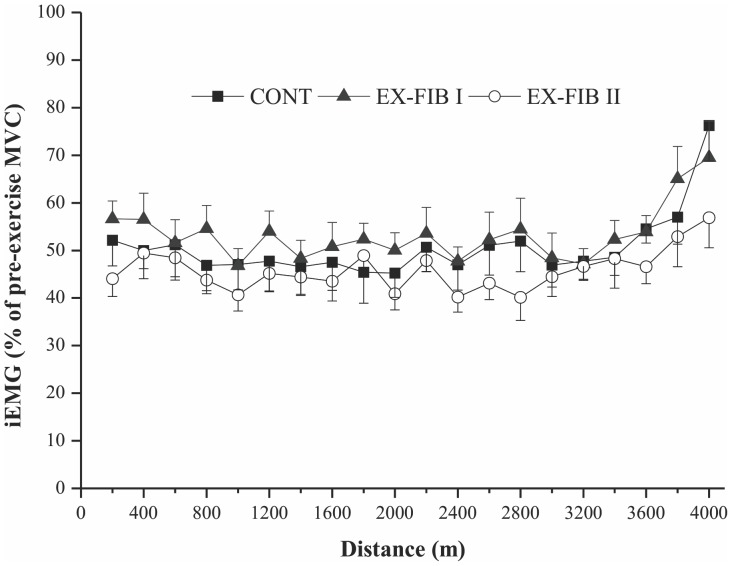
Mean and SEM for normalized integrated electromyography (% maximal voluntary contraction) during the 4-km cycling time trial in control (CONT) and reduced muscle glycogen conditions. EX-FIB I and EX-FIB II: time trial performed after a type I or type II muscle fibers glycogen-lowering exercise protocol, respectively.

There was no main effect for condition or distance, or an interaction effect, for iEMG (P>0.05; figure 3). A main effect for condition (P<0.05) and time (P<0.05), and an interaction effect (P<0.05), was observed for [La] (Table 2). The [La] increased significantly with exercise in all three conditions, but the increase was less pronounced in EX-FIB II than in CONT (P<0.05). There were no differences between CONT and EX-FIB I or between EX-FIB II and EX-FIB I (P>0.05).

**Table 2:** Mean plasma lactate concentration at rest, immediately before and 1 min after the 4-km cycling time trial in the control and reduced muscle glycogen conditions.

	CONT	EX-FIB I	EX-FIB II
Rest (mmol·L^−1^)	1.4±0.3	1.0±0.4	1.0±0.4
Pre-TT (mmol·L^−1^)	1.4±0.6	1.0±0.3	1.1±0.4
Post-TT (mmol·L^−1^)	11.4±1.9#	9.5±2.7#	8.8±1.2#*

Values are mean ± SEM. Pre-TT: plasma lactate concentration immediately before the time trial. Post-TT: plasma lactate concentration 1 min after the time trial. CONT: control condition. EX-FIB I and EX-FIB II: time trial performed after a carbohydrate-manipulation protocol designed to predominantly reduce muscle glycogen content in type I or II fibers, respectively. # Significantly higher than Rest and Pre-TT (P<0.05). * Significantly lower than CONT (P<0.05).

## Discussion

The aim of this study was to analyze the influence of two different exercise protocols, designed to predominantly reduce muscle glycogen levels in either type I or type II fibers, on pacing and performance during a 4-km cycling TT. We had hypothesized that an exercise protocol designed to reduce muscle glycogen in type II fibers would impair PO at the beginning and last part of the TT, while an exercise protocol designed to reduce muscle glycogen of type I fibers would impair PO in the midpoint of the TT. The main findings of the present study were that both prior muscle-glycogen-reducing exercise protocols altered pacing strategy and energy system contributions during a 4-km cycling TT. As a consequence, overall performance was impaired after both exercise protocols. These results might be attributed to a more conservative PO distribution pattern adopted by the athletes in the reduced muscle glycogen conditions. A decrease in PO at the beginning and middle of the trial was observed in EX-FIB I, accompanied by a reduced P_aer_ throughout the trial. On the other hand, PO mean and plasma [La] accumulation were decreased in EX-FIB II. The iEMG remained unchanged throughout the TT in all three conditions.

A lower mean PO and a longer time to completion of the TT was observed in EX-FIB I and EX-FIB II compared with CONT. Qualitative inferences indicated that these changes were ‘very likely’ harmful and ‘likely’ when EX-FIB I and EX-FIB II were compared to CON, respectively, suggesting that the impairments in performance were meaningful. Using time-to-exhaustion tests, it has previously been reported that performance during high-intensity exercise (80–90% of VO_2peak_; 10–20 min) and supra VO_2peak_ exercise (115% of VO_2peak_; ∼3 min) is impaired if athletes start the trial after a muscle-glycogen-lowering exercise protocol [27]–[29]. Likewise, the results of the present study are also in accordance with earlier studies showing a reduction in performance when participants performed a cycling TT after an exercise protocol designed to reduce concomitantly the muscle glycogen content of both type I and II muscle fibers [2]–[4]. However, to the best of our knowledge, this is the first study to investigate these effects during a TT carried out after validated exercise protocols designed to reduce selectively glycogen content in specific muscle fibers (type I or II fibers).

The PO-versus-distance curve in CONT showed a classic initial fast start, followed by a gradual decline in PO until completion of approximately 80% (3.2 km) of the TT, when the PO increased again in the last part, i.e. a U-shaped curve. This pacing model has previously been shown in studies investigating pacing during a TT of similar distance and duration [30]. In contrast, participants adopted a more conservative pacing strategy at the beginning and during the middle of EX-FIB I. Previous studies demonstrated that pacing at the beginning and middle [3] of a TT is affected negatively by the prior reduction in muscle glycogen stores. The results of the present study confirm our first hypothesis that an exercise protocol designed to predominantly lower muscle glycogen in type I muscle fibers impairs PO during the midpoint of a 4-km TT. On the other hand, our second hypothesis that previous depletion of the glycogen in the type II muscle fibers would impair PO at the beginning was not confirmed. In contrast, we found a reduction in PO at the beginning of the EX-FIB I trial when compared to CONT (figure 1). However, the aerobic contribution was systematically lower during EX-FIB I than CONT (figure 2A), suggesting that muscle glycogen depletion of type I fibers impaired aerobic metabolism from beginning of the exercise. It should be noted that a less aggressive start is accompanied by slower VO_2_ on-kinetics and a reduced aerobic contribution [3], [5], [31]. As any time lost during the first part of a time trial cannot be recovered later in the event [3], it could be suggested that the impaired performance in the EX-FIB I may have been partially due to the adoption of a suboptimal starting pacing.

In the middle part of the trial, participants maintained a more conservative pace in both reduced muscle glycogen conditions (figure 1). The PO in EX-FIB I was significantly lower than in CONT from 2.2 to 3.0 km, except for the 2.4-km interval. On the other hand, there was a significant reduction in the mean PO during the entire EX-FIB II trial when compared to CONT. It is noteworthy that, although the PO was slightly impaired in the middle of the trial, these differences were no longer apparent when separated into P_aer_ and P_an_ for EX-FIB II, but P_aer_ was systematically reduced during EX-FIB I (figures 2A and 2B).

The Post-TT [La] was significantly lower in EX-FIB II than in the CONT condition. An interaction effect indicates that the increase in [La] with exercise was less pronounced during EX-FIB II than during the CONT or EX-FIB I. A previous study has suggested that an exercise protocol designed to reduce glycogen from both type I and II muscle fibers reduces the anaerobic capacity by close to 20% during a single high-intensity (175 to 400 W) square-wave exercise [32]. In the present study, a significant reduction in plasma [La] accumulation during EX-FIB II suggests that selective muscle glycogen depletion of type II muscle fibers may produce a slightly reduce glycolysis during the exercise. However, overall P_an_ was not affected (∼9% of reduction, not significant), suggesting that selective muscle glycogen depletion may not produce a reduction in the anaerobic metabolism of the same magnitude as when both types of fibers are depleted concomitantly [32].

The iEMG of the vastus lateralis did not parallel the changes in PO throughout the trial. Specifically, iEMG remained unaltered throughout the trial (figure 3). We [33], [34] and others [35] have observed a similar behavior during a 4-km cycling TT. Interestingly, we also did not find significant differences in iEMG between the conditions despite a gradual decline in PO in both EX-FIB I and EX-FIB II. While iEMG cannot provide a measure of the specific recruitment of type I and type II muscle fibers during nonstationary conditions such as a TT, the iEMG signal may indicate a general muscle fiber recruitment profile. Our results therefore indicate that the muscle fiber recruitment pattern may not have been altered with muscle glycogen depletion of either type I or type II muscle fibers. It should be mentioned, however, that interpretation of the iEMG signal during variable external PO as in a TT is difficult and not free of misinterpretation [13]. The surface iEMG may not be sufficiently sensitive to measure small changes in muscle activation. In addition, the surface iEMG signal quantifies both motor unit recruitment (i.e. the number of motor units activated) and the firing rates of the activated motor units, reflecting together the muscle activation [13]. Currently, it is not possible to divide the signal to assess these two phenomena separately. Other concerns that may affect iEMG analysis include: 1) surface EMG amplitude cancellation; 2) the stability of neuromuscular propagation and; 3) fatigue related reflex inhibition (reflex effects in the spinal cord) [13]. Therefore, caution is warranted when interpreting iEMG data during a cycling TT.

Finally, we found that although the overall TT performance was impaired in both reduced muscle glycogen conditions, force production (i.e. maximal voluntary contraction) prior to the TT was similar between the three conditions. These results highlight the task-specific nature of fatigue and indicate that the mechanism(s) that dictate task failure during a TT may not necessarily be the same as those influencing pre-exercise maximal voluntary contraction performances. In other words, the specific mechanism responsible for performance reduction may be exercise task dependent [3], [36].

A possible limitation of the present study is that we did not use a gold-standard technique (e.g., muscle biopsy) to quantify the muscle glycogen content. However, the muscle biopsy technique is invasive and could have interfered directly with the athletes’ pacing and overall performance. Furthermore, the effectiveness of the selective muscle glycogen-lowering exercises used in this study is well established [8]–[10]. Gollnick et al. [8] found a reduced muscle glycogen content in almost all type I fibers after 3 hours of a constant-load exercise at 30% PPO. On the other hand, in the protocol (10 sets of 1 min at 120% PPO) used by Thomson et al. [9], almost all type II fibers had their muscle glycogen content discernibly reduced. Later, Carter et al. [10] applied these two validated protocols in their study and found that approximately 94% of type I fibers had their muscle glycogen depleted after the prolonged-exercise protocol, whereas approximately 99% of type II fibers had their muscle glycogen depleted after the high-intensity protocol. An additional potential limitation is that the time for recovery of muscle glycogen stores in type I and type II muscle fibers may have been different. However, because we used a short recovery period between the glycogen-depleting protocol and the main TT (∼12 h), and there were only two intervening meals, one of them two hours before the TT, a significant muscle glycogen replenishment is unlikely. Despite the total work during the glycogen-depleting task differing between the trials, no significant difference was found between pre-exercise maximal voluntary contractions, suggesting no influence of the muscle glycogen depletion protocol on pre-exercise neuromuscular fatigue. Taken together, although we did not directly measure muscle glycogen content, the results of these previous studies provide sufficient evidence for the validity of these protocols for use in applied research.

## Conclusion

The results of the present study suggest that prior, prolonged, low-intensity exercise (designed to reduce glycogen of type I muscle fibers) was associated with impairment in PO at the beginning and middle of a 4-km TT, and this was accompanied by a reduction in the aerobic contribution. On the other hand, the prior, short, high-intensity exercise (designed to reduce glycogen of type II muscle fibers) impaired the PO and reduced the plasma [La] accumulation. Irrespective of the inclusion of either a prior prolonged, low-intensity exercise or short, high-intensity exercise, an impairment in overall performance of a middle-distance cycling TT was evident, but the pacing adopted, as well as energy system alterations, occurred in a specific way. To the best of our knowledge, this is the first study demonstrating that the distribution of energy expenditure during a middle-distance cycling TT is dependent of the intensity and duration of the exercise carried out in the evening before.
